# Skeletal Muscle-Released Extracellular Vesicles: State of the Art

**DOI:** 10.3389/fphys.2019.00929

**Published:** 2019-08-09

**Authors:** Sophie Rome, Alexis Forterre, Maria Luisa Mizgier, Karim Bouzakri

**Affiliations:** ^1^CarMeN Laboratory (UMR INSERM 1060/INRA 1397, Lyon 1), Lyon-Sud Faculty of Medicine, University of Lyon, Pierre-Bénite, France; ^2^Department of Microbiology and Immunology, Stanford University School of Medicine, Stanford, CA, United States; ^3^UMR DIATHEC, EA 7294, Centre Européen d’Etude du Diabète, Université de Strasbourg, Strasbourg, France

**Keywords:** exosomes, microparticles, skeletal muscle (myotubes), extracellular vesicles, organ cross-talks

## Abstract

All cells export part of their intracellular content into the extracellular space through the release of various types of extracellular vesicles (EVs). They are synthetized either from the budding of the plasma membrane [i.e., microparticles (MPs, 150–300 nm size)] or from the late endosomes in which intraluminal vesicles progressively (ILVs) accumulate during their maturation into multivesicular bodies (MVBs). ILVs are then released into the extracellular space through MVB fusion with the plasma membrane [i.e., exosomes (50–100 nm size)]. In the context of metabolic diseases, recent data have highlighted the role of EVs in inflammation associated with pancreas dysfunction, adipose tissue homeostasis, liver steatosis, inflammation, and skeletal muscle (SkM) insulin resistance (IR). Among these insulin-sensitive tissues, SkM is the largest organ in human and is responsible for whole-body glucose disposal and locomotion. Therefore, understanding the contribution of SkM-EVs in the development of diabetes/obesity/dystrophy/,-related diseases is a hot topic. In this review, we have summarized the role of SkM-EVs in muscle physiology and in the development of metabolic diseases and identify important gaps that have to be filled in order to have more precise information on SkM-EVs biological actions and to understand the functions of the different subpopulations of SkM-EVs on the whole-body homeostasis.

## Introduction

Skeletal muscle (SkM) is the largest organ in the human body. It is responsible for whole-body glucose and energy homeostasis, locomotion, and serves as body protein pool. It is a highly adaptable tissue responding to numerous environmental conditions (e.g., physical activity/sedentarity) and physiological challenges (e.g., nutrition, chronic inflammation) by changing fiber size and composition. These modifications are associated with the secretion of myokines into the extracellular milieu capable of modulating homeostatic adaptations in other peripheral organs (e.g., pancreas, adipose tissue, and bone) (Plomgaard et al., 2012; Guo et al., 2017; Leal et al., 2018; Lee and Jun, 2019) or involved in the process of myogenesis (Henriksen et al., 2012). During the last decade, it has been shown that muscle cells also release extracellular vesicles (EVs) into the extracellular milieu, which represent new paracrine and endocrine signals that have modified our conceptual basis to explain how muscles communicate to other organs (Guescini et al., 2010; Le Bihan et al., 2012; Romancino et al., 2013; Forterre et al., 2014a). In this review, we summarize the knowledge about SkM-EV contents (protein/nucleic acids/lipids) and on the role of SkM-EVs on muscle physiology and on the development of metabolic diseases. We also identify important questions that have to be elucidated in order to have more precise information on SkM-EVs biological actions and to understand the functions of the different subpopulations of SkM-EVs on the whole-body homeostasis.

### Skeletal Muscle Cells Release Various Types of Extracellular Vesicles

**Figure 1:**
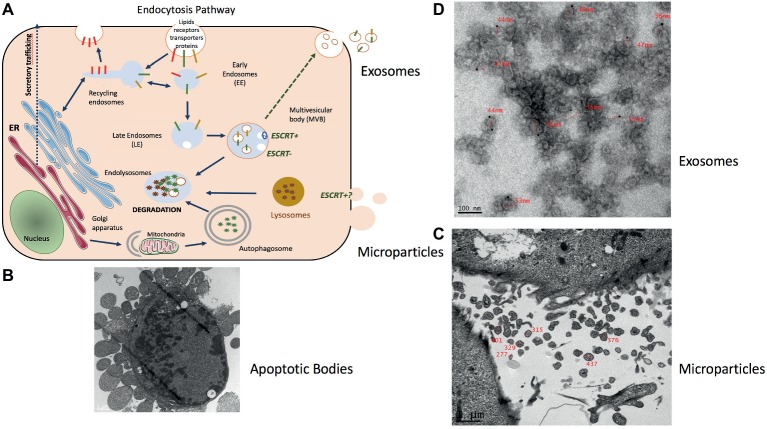
Representation of muscle-release extracellular vesicles (EVs). **(A)** Exosomes are formed in late endosomes called multivesicular endosomes (MVBs), containing internal vesicles (ILVs) that pack and store molecules in membrane-bound structures. Endosomes are an intermediate compartment between the plasma membrane where endocytosis takes place and lysosomes where these molecules are degraded. MVB biogenesis can occur with the ESCRT machinery (ESCRT^+^) or without (ESCRT^−^) (Hessvik and Llorente, 2018). MVBs rich in lysobisphosphatidic acid (LBPA) but low in cholesterol migrate toward lysosomes and fuse with them. Those rich in cholesterol but low in LBPA migrate to the plasma membrane to fuse and release their ILVs as exosomes (Laulagnier et al., 2005; Colombo et al., 2014). **(B)** Electron microscopy showing the release of apoptotic bodies from an apoptotic C2C12 myoblast (personal data from S. Rome) (scale = 1 μm). **(C)** Microparticles (MPs) represent a heterogeneous population of small plasma membrane vesicles (Morel et al., 2011). Electron microscopy of human myotubes showing the release of MPs from the plasma membrane (scale = 1 μm) (personal data from S. Rome). **(D)** Due to their small size exosome-like vesicles (ELVs) can be visualized only through electron microscopy. ELVs released from *quadriceps* explants labeled with anti-CD81 gold particles (personal data from S. Rome).

All cells export part of their content into various types of extracellular vesicles (EVs) (Figure 1A). They are synthetized either from the budding of the plasma membrane [i.e., microparticles (MPs), 150–300 nm size)] or from the maturation of late endosomes in which intraluminal vesicles (ILVs) progressively accumulate to form the multivesicular bodies (MVBs). ILVs are then released into the extracellular space through exocytosis of MVB [i.e., exosomes (EXO), 50–110 nm size] (Johnstone et al., 1991; Colombo et al., 2014). Larger vesicles are also formed during cell apoptosis when the cell cytoskeleton breaks up and induces the membrane to bulge outward (i.e., apoptotic bodies of 300–2,000 nm size) (Figure 1B; Caruso and Poon, 2018). It has been shown that apoptotic bodies are involved in tissue repair and angiogenesis during atherosclerosis (Zernecke et al., 2009). MPs are involved in inflammation, coagulation, vascular function, and sepsis (Boulanger, 2010; Reich et al., 2011; Das, 2019). They also release proteases for extracellular matrix degradation (Lozito and Tuan, 2012) and promote oncogene propagation among subsets of cancer cells (Al-Nedawi et al., 2008). Shedding of membrane MP is also observed in normal conditions, e.g., MPs released from normal endothelial cells are implicated in angiogenesis. EXO are involved in cell homeostasis to eliminate unwanted and toxic cellular compounds (proteins, lipids, and nucleic acids) (Johnstone et al., 1991; Desdin-Mico and Mittelbrunn, 2017). As these types of vesicles do not originate from the same cellular compartment their composition (lipid/nucleic acids and proteins) are different (Crescitelli et al., 2013; Durcin et al., 2017; Wei et al., 2017; Tucher et al., 2018).

**Figure 2:**
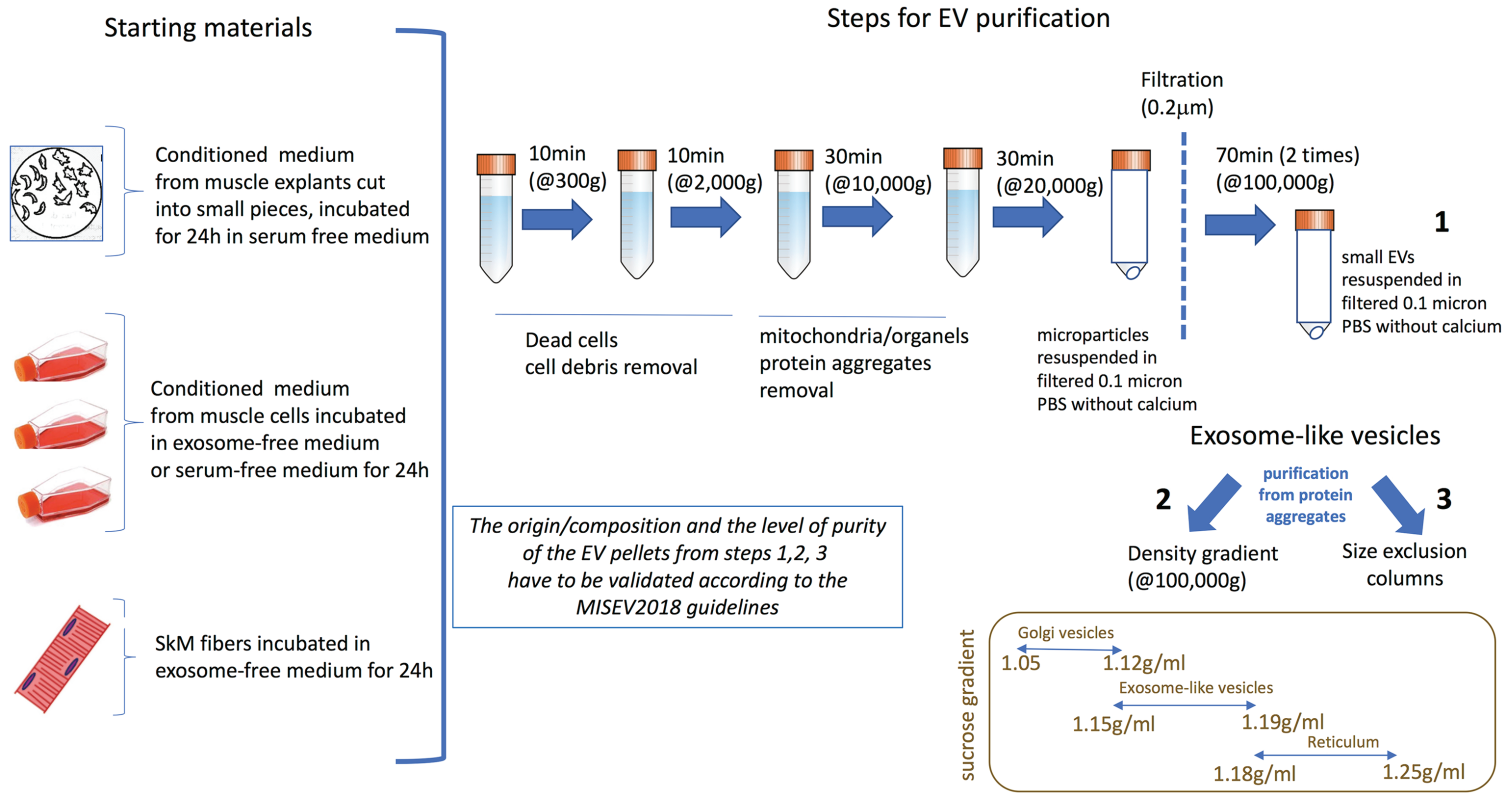
Currently used protocols to purify ELVs from skeletal muscle (SkM) cells conditioned medium, SkM explants, or myofibers. The origin/composition and the level of purity of the EV pellets from steps 1–3 have to be validated according the MISEV2018 guidelines (Thery et al., 2018).

To isolate these different types of EVs from muscle cell conditioned medium (Figures 1C,D) differential centrifugations are performed as described in Thery et al. (2006) (Figure 2). After MP removal, and in order to enrich the preparation in small EVs, the supernatant is passed through a 0.22-μm filter and then is ultracentrifugated to collect small EVs enriched in exosomes. Sucrose or iodixanol gradients, or size exclusion chromatography with open columns are recommended to remove protein aggregates that may precipitate with the small EVs (Thery et al., 2018; Jeppesen et al., 2019). It has to be noticed, however, that these different methods have not been compared for muscle-released EVs and may result in the selection or enrichment of specific EV subpopulations. As it is difficult to avoid contaminations of exosomes with other small vesicles, (i.e., small MP or small apoptotic bodies) and because no highly extensive vesicle purification was performed in published studies working on isolated EVs from SkM cells, SkM explants, or myofibers, we will use the term of exosome-like vesicles (ELV) in this review to consider the small EV subpopulation’s heterogeneity.

The process of myogenesis involves a first step in which myoblasts proliferate until they reach confluence, then fuse to produce multinucleated myotubes which do not proliferate anymore. Nuclei are located at the periphery of the myofiber and the other organelles are squeezed in between myofibrils or between myofibrils and the plasma membrane. How myotubes and myoblasts, which are very different type of cells, generate these small ELVs has not been yet investigate. It has been described that myoblasts produce small ELVs (ELV-MB) (Guescini et al., 2010; Forterre et al., 2014a,b; Sork et al., 2018) likely reflecting the intense macromolecule/RNA/lipids turn-over that occurs during cell proliferation (Johnstone et al., 1991; Desdin-Mico and Mittelbrunn, 2017). Myotubes also release small ELVs (ELV-MT) (Figure 1D; Le Bihan et al., 2012; Romancino et al., 2013; Forterre et al., 2014a,b) and the presence of ELVs was also demonstrated in dispersed mouse myofibers from the mouse hindlimb muscles (De Gasperi et al., 2017). Romancino et al. (2013) have suggested that ELV-MB and ELV-MT may have different origins and that ELVs from MT would be produced mainly through the budding of the plasma membrane. In their study, loss of Alix protein involved in ILV biogenesis in MVBs (Sun et al., 2015) resulted in alteration of membrane budding, a decrease in the number of ELV released and a modifications of ELV-MT composition (of note, in this study the EV pellet was not filtered at 0.22 μm; thus, the authors may have worked with a pool of MPs and ELVs) (Romancino et al., 2013). Interestingly, the involvement of Alix for EV release seems to occur only in muscle cells as the knockdown of Alix did not affect the number of ELVs released from human liver stem-like cells (Iavello et al., 2016).

**Figure 3:**
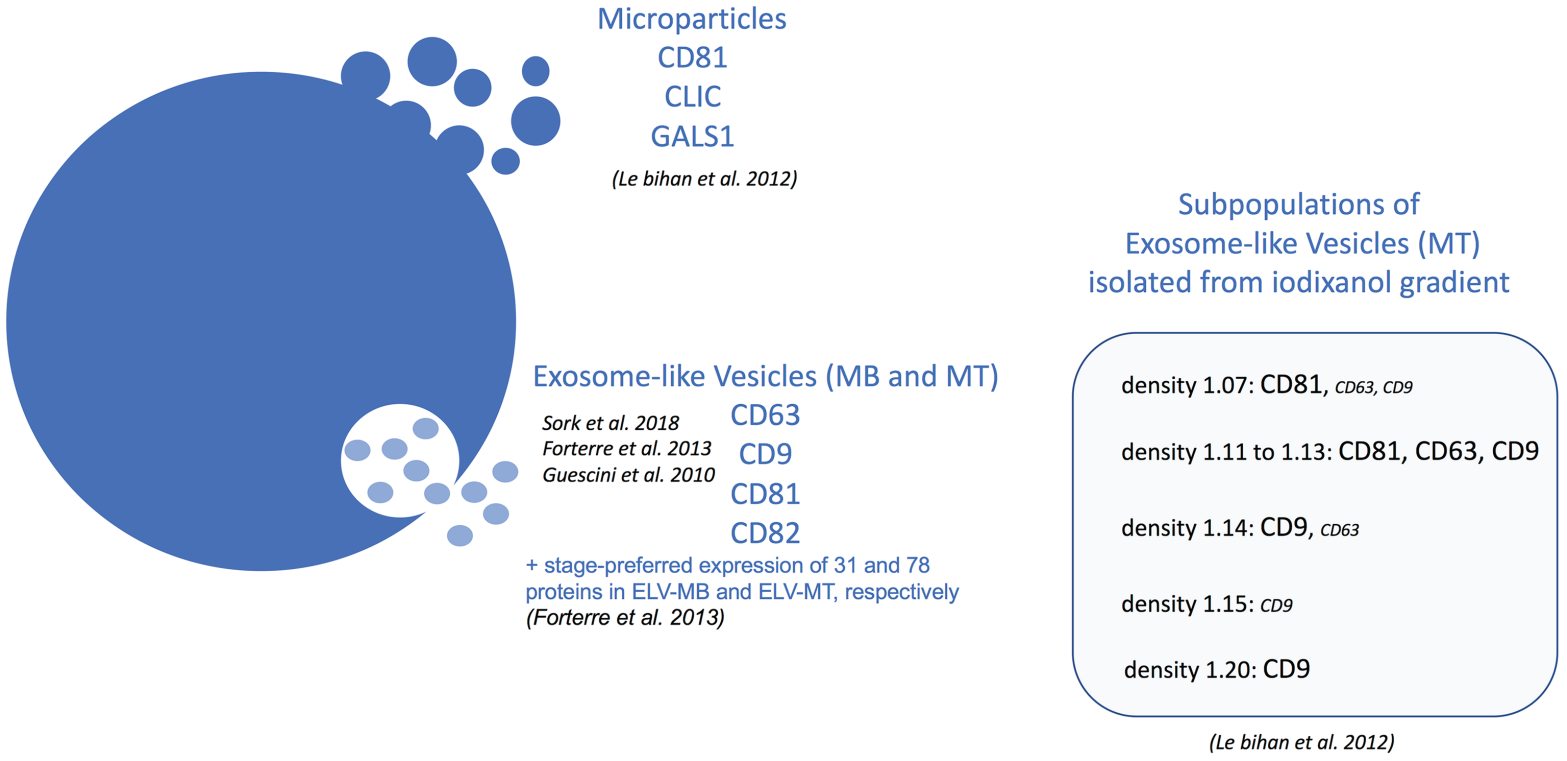
Summary of all vesicles identified in the conditioned-medium from skeletal muscle (SkM) cells based on the detection of specific subsets of tetraspanins by proteomics or Western-blot.

MPs released from human MT were enriched in tetraspanin CD81 and in CLIC1 and galectin-1 (LGALS1) proteins and did not contain tetraspanins CD63, CD82, and CD9 which were found associated with ELVs from human MT (Figures 1D, 3; Le Bihan et al., 2012). Proteomic analyses indicated that ELV-MT contain few of the endosomal/lysosomal CD63 tetraspanin compared with CD81 or CD9 tetraspanin (Forterre et al., 2014a), known to be also expressed at the plasma membrane^1^. Western blot quantifications also showed that CD81 was more enriched in ELV-MT than in ELV-MB (Forterre et al., 2014a) and that Alix protein level was significantly enriched in ELVs released from myotubes than from myoblasts (Romancino et al., 2013). Taken together, these data highlight two pathways for ELV release from muscle cells (i.e., for ELVs and MPs, respectively) and suggest that the ratio between ELVs and MPs may vary between proliferation and differentiation. The contribution of these two types of vesicles in muscle physiology has never been fully characterized, and their specific biological functions on recipient cells are not known.

Beside EVs biogenesis’ variability resulting in the release of different vesicle subpopulations, recent data from other cell types indicated that when sucrose gradient purifications were performed, small ELVs might be composed of different subpopulations. For instance, ELVs released from colon carcinoma cell line LIM1863 (Tauro et al., 2013), human dendritic cells (Kowal et al., 2016), human mast cell HMC-1 (Crescitelli et al., 2013), and RBL-2H3 (Laulagnier et al., 2005) are a mixture of two subpopulations of small EVs with distinct densities when separated on sucrose or iodixanol gradients. In addition, they are distinguished by their total RNAs, proteins, and lipids content too. For mesenchymal stem cells, at least three populations with specific protein and lipid contents were identified (Lai et al., 2016). Based on the recent study of Jeppesen et al. (2019), it is likely that these two populations represent exosomes and small MPs. For human muscle cells, iodixanol gradients have been performed with ELV-MT and resulted in the identification of five potential ELV-MT subpopulations with specific tetraspanin enrichments (Figure 3; Le Bihan et al., 2012). Interestingly, mitochondrial DNA (Guescini et al., 2010), histones, and nuclear proteins were also found in ELV-MB (Guescini et al., 2010; Forterre et al., 2014a; Sork et al., 2018) and ELV-MT (Le Bihan et al., 2012; Forterre et al., 2014a). Although we cannot exclude the presence of contaminations with small apoptotic bodies which are formed during myoblast apoptosis, it has to be noticed that RNA/proteins of both nuclear and mitochondrial origins have been consistently identified in ELVs released from other cell types suggesting that SkM may also release EVs originated from different MVB subpopulations (Vesiclepedia: http://microvesicles.org/index.html). Indeed, it has been shown that cells contain different MVB-like structures and some of MVBs contain vesicles of different sizes (small vesicles can be inside larger ones) or filled with electron-dense material (Zabeo et al., 2017). Interestingly, it was shown that mitochondria were able to release small vesicles from their membranes to deliver specific mitochondrial contents into late endosome/MVBs (Soubannier et al., 2012; Sugiura et al., 2014; Cadete et al., 2016). In addition, the formation of nuclear vesicles was also reported as non-canonical pathway for mediating nucleo-cytoplasmic transport of ribonucleoprotein particles in *Drosophila* larvae muscle (Speese et al., 2012). All these data suggest a high level of material exchange between different types of MVB-like structures, which could result in the release of a heterogeneous population of SkM-ELVs and may explain the diversity of their protein contents. In line with this hypothesis, a new pathway was recently described to explain the presence of dsDNA and histones in small EVs. It would involve MVB and autophagy and the formation of an amphisome that, after fusion with the plasma membrane, would release dsDNA and histones in the extracellular space, as an exosome-independent mechanism (Jeppesen et al., 2019). It is now of utmost importance to characterize SkM-EVs subpopulations, to understand their cellular origin, and to determine whether they may have distinct biological activities on recipient cells (Lasser et al., 2018).

### Composition of Skeletal Muscle-Released Extracellular Vesicles

#### Proteins

**Figure 4:**
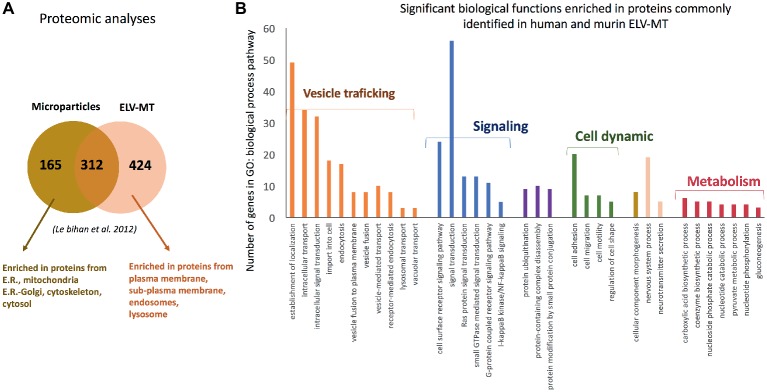
**(A)** Proteomic analyses identified specific subsets of proteins in MPs and ELVs released from differentiated human SKM cells [full list of proteins are in Le Bihan et al. (2012)], protein are identified from protein fragments). **(B)** Significant GO biological functions enriched in genes commonly identified C2C12 myotubes- and primary human myotubes-ELVs isolated from conditioned medium. Proteomic data are from Forterre et al. (2014a) and Le Bihan et al. (2012) and were analyzed with PANTHER version 11 (Mi et al., 2017).

**Figure 5:**
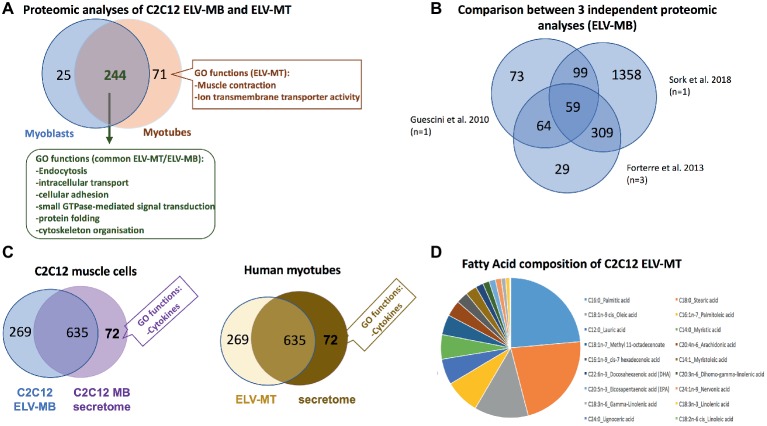
**(A)** Overlap between the number of proteins from C2C12 ELV-MB and ELV-MT (see Forterre et al., 2014a for a full list of proteins, proteins are identified from protein fragments). **(B)** Overlap between three independent protomic analyses from C2C12 ELV-MB. **(C)** Left: overlap between the protein content of ELV-MB and the secretome from C2C12 myoblasts (Forterre et al., 2014a); right: overlap between the protein contents of ELV-MT and the secretome from human differentiated MT (Le Bihan et al., 2012). Only significant GO functions between the two sets of proteins are indicated. **(D)** Fatty acid composition of ELV-MT from C2C12 (Aswad et al., 2014).

Proteomic analyses have identified two specific subsets of proteins for MPs and ELVs, released in the conditioned medium from human myotubes (Figure 4A; Le Bihan et al., 2012). MPs were enriched in genes coding for proteins involved in “protein synthesis, folding, and trafficking,” “RNA post-transcriptional modifications,” “translation modifications,” and “amino acid metabolism.” They were mainly cytosolic proteins, located in “endoplasmic reticulum,” “Golgi vesicles,” “mitochondria,” and “cytoskeleton.” Compared to MPs, human ELV-MT were enriched in proteins for “free-radical scavenging” and “cell-to-cell signaling and interactions.” Conversely to MP proteins, ELV-MT proteins were localized in “subplasma-membrane cytoplasmic vesicles,” “endosomes,” “lysosome,” and “plasma membrane” (Le Bihan et al., 2012), further suggesting that SkM would release small vesicles also through the budding of the plasma membrane (Romancino et al., 2013). These comparative proteomic analyses indicated that ELV-MT proteins are mainly those endocytosed and delivered into endosomes for incorporation into MVBs or for degradation into lysosomes. The selection process involved in the delivery of some membrane proteins to ILVs of MVBs for destruction while others remain outside is not well understood. It has been found that ubiquitination was necessary the partitioning of mainly, but not all, membrane proteins into invaginating MVBs (Reggiori and Pelham, 2001; Stringer and Piper, 2011). In this way, endosomes/MVBs regulate the composition of plasma membrane and thus play a pivotal role in a vast array of biological functions. For instance, the endosomal system functions like a digital-analogue computer that regulates the specificity and robustness of the insulin signaling response in the SkM (i.e., insulin induces translocation of key signaling proteins into endosome) (Balbis et al., 2000; Jethwa et al., 2015). The presence of proteins involved in gluconeogenesis, pyruvate metabolism, and biosynthesis of carboxylic acids (e.g., TPI1, PGK1, GAPDH, GPI, ENPP1, and ACLY) also suggests that ELV-MT release could represent an important pathway for the turnover of proteins involved in the regulation of SkM metabolism (Figure 4B). Gene enrichment analyses of the proteins commonly identified in ELV-MT from human (Le Bihan et al., 2012) and murine (Forterre et al., 2014a) SkM cells further confirmed that ELV-MT are enriched in proteins involved in exosome formation and intracellular trafficking (e.g., endocytosis, vesicle fusion, and transport, and signaling pathways involved in vesicle cellular localization) (Figure 4B). Interestingly, the majority of the proteins identified in ELV-MB was also found in ELV-MT (Figure 5A). This result indicates that although the organization of the cellular organelles and the plasma membrane of myoblasts changes dramatically during differentiation consequently to the formation of a single functional unit, SkM-ELV composition remains quite constant. This result further supports the concept that proteins sorting into ELVs appears to be selective and almost the same subset of proteins is exported whatever type of cell considered. Figure 5B shows the overlapping between three independent proteomic analyses from C2C12 myoblasts and the 59 proteins commonly identified are listed in Table 1. Of note, only CD63 was commonly identified among all tetraspanins (Figure 3) likely reflecting the protocol variabilities for SkM EV purifications (Jeppesen et al., 2019).

**Table 1:** Proteins identified in 3 independant proteomic analyses from C2C12 ELV-MB.

Gene symbols	Names
Aldoa	aldolase A, fructose-bisphosphate
Ldha	lactate dehydrogenase A
Gapdh	Glyceraldehyde 3-phosphate dehydrogenase
Anxa1	annexin A1
Anxa2	annexin A2
Anxa3	annexin A3
Anxa4	annexin A4
Anxa5	annexin A5
Anxa6	annexin A6
Arhgdia	Rho GDP dissociation inhibitor (GDI) alpha
Cct3	chaperonin containing Tcp1, subunit 3 (gamma)
Cct4	chaperonin containing Tcp1, subunit 4 (delta)
Cct5	chaperonin containing Tcp1, subunit 5 (epsilon)
Cct6a	chaperonin containing Tcp1, subunit 6a (zeta)
Cct8	chaperonin containing Tcp1, subunit 8 (theta)
Cd63	CD63 antigen
Clic1	chloride intracellular channel 1
Cltc	clathrin, heavy polypeptide (Hc)
Des	desmin
Eef1a1	eukaryotic translation elongation factor 1 alpha 1
Eef2	eukaryotic translation elongation factor 2
Ehd1	EH-domain containing 1
Ehd4	EH-domain containing 4
Eif4a1	eukaryotic translation initiation factor 4A1
Ezr	ezrin
Fn1	fibronectin 1
Gnb2	guanine nucleotide binding protein (G protein), beta 2
Hspa8	heat shock protein 8
Hspb1	heat shock protein 1
Igsf8	immunoglobulin superfamily, member 8
Ipo5	importin 5
Itgb1	integrin beta 1 (fibronectin receptor beta)
Kpnb1	karyopherin (importin) beta 1
Msn	moesin
Myh9	myosin, heavy polypeptide 9, non-muscle
Pdcd6	programmed cell death 6
Pdcd6ip	programmed cell death 6 interacting protein
Psmc6	proteasome (prosome, macropain) 26S subunit, ATPase, 6
Ptgfrn	prostaglandin F2 receptor negative regulator
Ran	RAN, member RAS oncogene family
Rplp0	60S acidic ribosomal protein P0
Rps2	ribosomal protein S2
Rps3	Ribosomal Protein S3
Rps4x	ribosomal protein S4, X-linked
Rps8	ribosomal protein S8
Rpsa	ribosomal protein SA
Tpi1	triosephosphate isomerase 1
Tpt1	tumor protein, translationally-controlled 1
Tubb2b	tubulin, beta 2B class IIB
Tubb3	tubulin, beta 3 class III
Tubb5	tubulin, beta 5 class I
Tubb6	tubulin, beta 6 class V
Uba1	ubiquitin-like modifier activating enzyme 1
Vcl	vinculin
Vcp	valosin-containing protein
Vim	vimentin
Ywhae	tyrosine 3-monooxygenase/tryptophan 5-monooxygenase activation protein, epsilon polypeptide
Ywhaq	tyrosine 3-monooxygenase/tryptophan 5-monooxygenase activation protein, theta polypeptide
Ywhaz	tyrosine 3-monooxygenase/tryptophan 5-monooxygenase activation protein, zeta polypeptide

In order to determine the contribution of the ELV-associated proteins among the set of proteins isolated from muscle cell secretome, a functional enrichment analysis was performed by comparing the set of secreted proteins from C2C12 myoblasts (Henningsen et al., 2010) with the set of proteins identified from ELV-MB (Figure 5C; Forterre et al., 2014a). None of the GO categories previously found as significantly enriched in ELV proteins (Figure 4B) was identified in the 635 secreted proteins. By contrast, the C2C12 secretome was significantly enriched in GO term “cytokines.” A similar result was obtained for human muscle cells (Le Bihan et al., 2012). These data indicated thus that SkM cell uses distinct pathways for the release of distinct protein subsets with likely distinct biological actions. One exception is the protein C1QTNF3 (Cartonectin) found in ELV-MT (Forterre et al., 2014a). Cartonectin is a new cytokine, paralog of adiponectin contained as a transmembrane protein in adipocyte-released EVs and in EVs isolated from serum (Phoonsawat et al., 2014). The function of this family of cytokines in ELVs is presently unknown.

#### Lipids

Lipids are essential constituent of ELV membranes, and several studies have described that specific lipids are enriched in ELVs compared with the parent cells (Skotland et al., 2017), i.e., a cholesterol, sphingomyelin, phosphatidylcholine, phosphatidylserine, phosphatidylethanolamine, and diacylglycerol. This specific lipid enrichment, their membrane high protein/lipid ratio and asymmetric distribution are associated with a higher membrane rigidity in ELVs in comparison with parent cells (Record et al., 2018). Lipidomic and proteomic analyses on ELVs and MPs released from three different cell lines have indicated that lipidomes of ELVs, MP and secreting cells were more similar to each other than their proteomes are (Haraszti et al., 2016). For muscle cells, only one study has determined the fatty acids (FA) composition of ELV-MT. As shown on Figure 5D, ELV-MT are enriched in palmitic acid, stearic acid, oleic acid, palmitoleic acid, and lauric acid compared to others FA (Aswad et al., 2014). These fatty acids are used for energy production or to produce phospholipids for phospholipid bilayers out of all cell membranes. Interestingly, ELVs released from C2C12 myotubes treated with palmitate were enriched in palmitate compared to BSA-treated cells (Aswad et al., 2014). As accumulation of intracellular fatty acids is associated with insulin resistance and/or an impaired glucose metabolism, the release of ELVs may represent a form of protection for the cell, to prevent accumulation of intracellular fatty acids.

#### RNA

**Figure 6:**
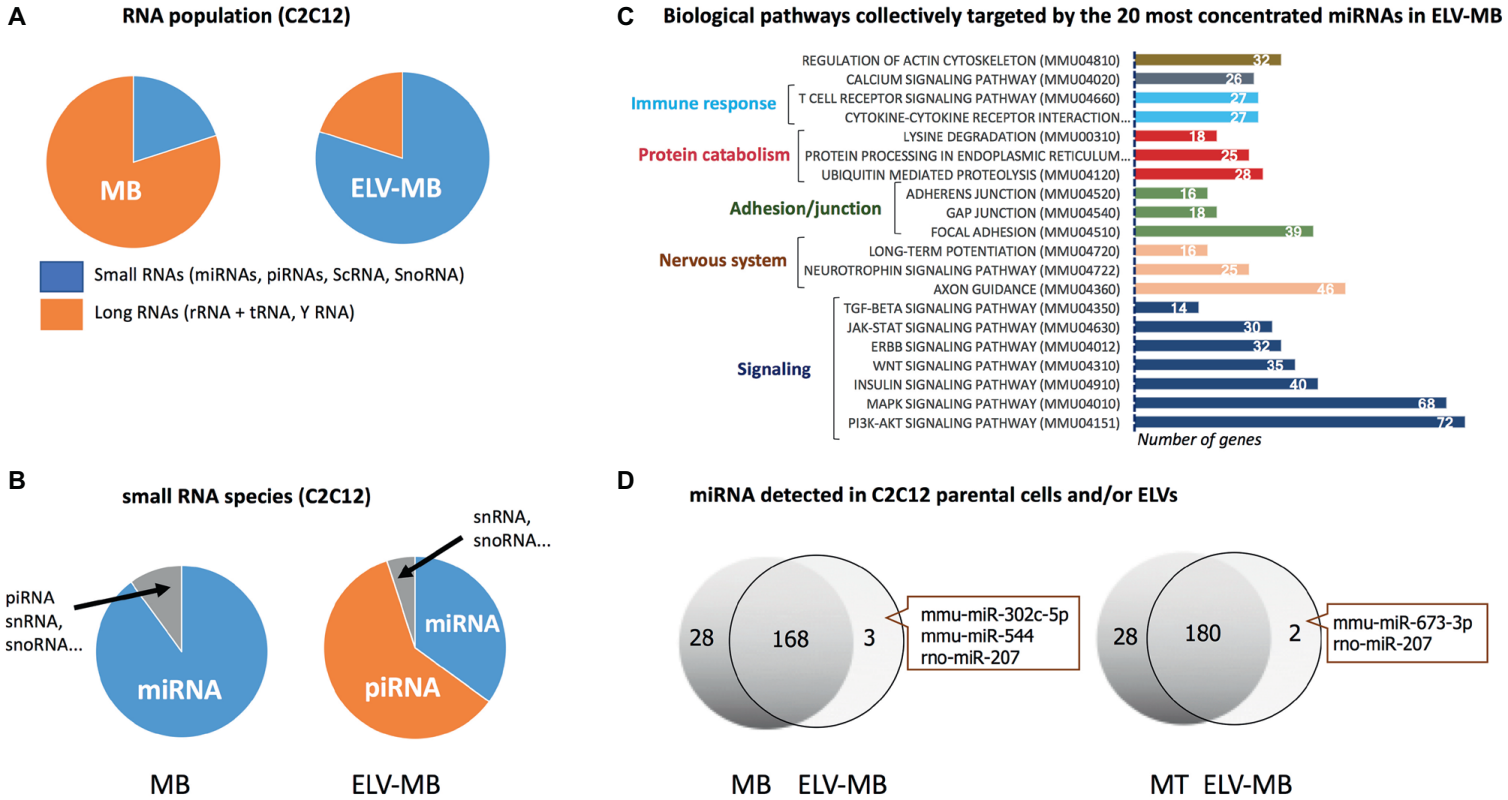
**(A–C)** were built from the sequencing data published in Sork et al. (2018). **(A)** Percentage of small and large RNAs in C2C12 myoblasts and their released ELV-MB. **(B)** Number small RNA species in C2C12 myoblasts vs. ELV-MB expressed as percentages. **(C)** Functional enrichment analyses of the predicted target genes from ELV-MB miRNAs. Target predictions and the identification of significant biological pathways were performed with DIANA-microT-CDS and mirPath v.3 (http://snf-515788.vm.okeanos.grnet.gr). **(D)** Overlap between the miRNA species found in muscle cells and their respective ELVs (*n* = 3). The same quantity of total RNA was used as starting material for qRT-PCR (Forterre et al., 2014b).

ELV preparations from various cell types have demonstrated that they contain distinctive repertoires of RNA populations including transcripts (Valadi et al., 2007; Skog et al., 2008) and non-coding RNAs, i.e., long non-coding RNA, microRNAs (miRNAs), piwi protein interacting RNA (piRNA), small nuclear RNA (snRNA), small nucleolar RNA (snoRNA), small Cajal body-specific RNA (scaRNA), circular RNA (circRNA), sc RNA Y, natural antisense RNA (asRNA), ribosomal RNA (rRNA), and vault RNA (vRNA) (Nolte-’t Hoen et al., 2012). Interestingly, while cellular RNAs are enriched in full-length long RNA species, ELVs are enriched in shorter RNA species (≤200 nucleotides) including short transcripts, fragmented mRNAs (Batagov and Kurochkin, 2013), and small RNAs (i.e., miRNA, snoRNA, snRNA, Y RNA, and vault RNA). As the sequencing of these different populations often rely on separate libraries the direct comparisons between them are not always straightforward, but these analyses clearly indicated a differential profile of ncRNA subsets between parental cells and EVs (Guduric-Fuchs et al., 2012). In the case of SkM cells, it was shown that C2C12 ELV-MB were also enriched in small RNAs subsets compared with C2C12 parental cells (Figure 6A; Sork et al., 2018). Only the small RNA population has been further analyzed (Forterre et al., 2014b; Sork et al., 2018; Xu et al., 2018), and was found highly enriched in piRNAs and miRNAs (Figure 6B; Sork et al., 2018). For the majority of miRNAs, a good correlation was observed between miRNA levels in ELV-MB vs. MB in two independent studies (Forterre et al., 2014b; Sork et al., 2018), and between ELVs released from myofibers vs. myofibers (De Gasperi et al., 2017), suggesting that export of miRNAs into ELVs would permit to control their intracellular concentrations. *In silico* predictions of the most concentrated miRNAs in ELV-MB (Sork et al., 2018) indicated that they could collectively regulate important cellular pathways for muscle physiology, i.e., signaling pathways involved in the regulation of muscle mass (PI3K-Akt, insulin, MAPK, TGF-beta, proteolysis, and calcium), in the neuromuscular junction, in immune response, and in calcium signaling and cytoskeleton (Figure 6C). Interestingly, all studies reported also that individual miRNAs deviate from this relationship, i.e., some miRNAs expressed in muscle cells were not detected in ELV-MB (Forterre et al., 2014b) and/or ELV-MT (Figure 6D; Forterre et al., 2014b); other were found at higher rate in ELV-MB vs. myoblasts [miR-451, miR-6239, miR-6,240, miR-6236, miR-144, miR-223, miR-5112, miR-3062, miR-142a, and miR-2137 in Sork et al. (2018)]. Similarly, miR-720 was enriched in myofiber-derived ELVs compared with parent myofibers and inversely for miR-1 (De Gasperi et al., 2017). A third category was composed of miRNAs that were only found in ELVs and not detectable in the parental cells (Figure 6D). On the contrary, miR-147, miR-30b-3p, miR-467c, miR-615-3p, miR-669a, miR-677, miR-28a-3p, and miR-29-2-5p were never detected in C2C12-released ELV-MB or ELV-MT (Forterre et al., 2014b). Taken together, these data indicated that the packaging of some specific miRNAs into muscle-released ELVs is selective. How miRNAs are loaded into ELVs is poorly understood and may be resulting from different mechanisms, i.e., (1) the 3′ end uridylation of miRNA directs these small RNAs to sites of ELV biogenesis, while adenylation appears to have the opposite effect (Koppers-Lalic et al., 2014); (2) the association of miRNAs with RNA-binding proteins which are targeted to MVBs through post-translation modifications (i.e., sumoylation of the ribonucleoprotein hnRNPA2B1, which recognizes the GGAG motif in a subset of miRNAs is associated with miRNA loading into MVB) (Villarroya-Beltri et al., 2013); (3) the association of miRNAs with proteins involved in their synthesis and their function, which are loaded into ELVs [i.e., AGO2 (Gibbings et al., 2009) and the elements of the RISC-loading (silencing) complex (ALIX) (Iavello et al., 2016)]; (4) the association with YBX1 localized in P-bodies which are closely juxtaposed to MVBs. It is hypothesized that YBX1 may complex with miRNAs whose mRNA targets are not expressed. This association would result in their sorting into MVBs for export by ELVs (Shurtleff et al., 2016).

Unlike miRNAs, there was no correlation between ELV-MB and cellular concentrations in myoblast for piRNAs (Sork et al., 2018). C2C12 ELV-MB also contained tRNAs (Sork et al., 2018) and Y RNA (mainly RNY1) (Forterre et al., 2014b; Sork et al., 2018). The function of these non-coding RNA in muscle-released ELVs has not been yet identified but based on recently published data from other cell types; they could participate in the biological action of SkM-ELVs (Cambier et al., 2017).

Messenger RNAs were also detected by using DNA microarrays, in ELVs and MPs released from human SkM cells (Le Bihan et al., 2012). Respectively, 185 and 4,431 transcripts were identified in ELVs and MPs. Hierarchical clustering showed that the mRNA cargos of ELVs and MPs were more closely related to each other than to the expression pattern of the secreting myotubes. The abundance of the 185 ELV mRNAs correlated with their abundance in myotubes, indicating that myotubes release a specific subset of transcripts in ELVs, and not only the most concentrated one. ELVs were significantly enriched in transcripts encoding receptors and ionic channels, and regulators of transcription in particular zinc-finger transcription factors (Le Bihan et al., 2012).

### Biological Action of Skeletal Muscle-Released Extracellular Vesicles

**Figure 7:**
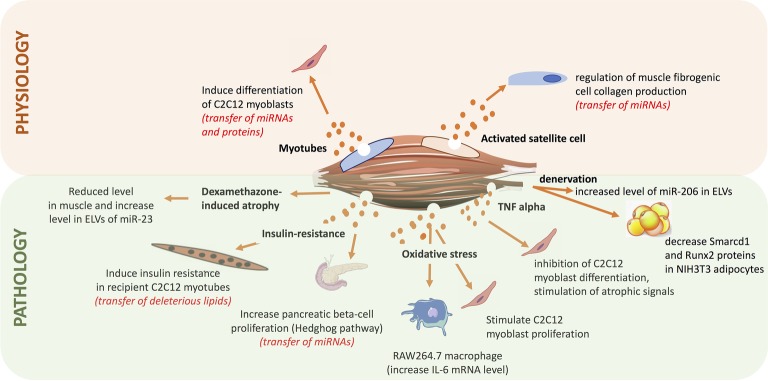
Summary of the roles of exosome-like vesicles released from skeletal muscle (SkM) cells, published so far.

Since the discovery that ELV cargos released from a specific cell type can be incorporated into various recipient cells (including a re-uptaking by the releasing cells) and by this way can regulate their fate (Valadi et al., 2007), numerous studies have demonstrated the involvement of EVs in health (i.e., embryogenesis and development, tissue homeostasis) and diseases (i.e., cancer metastasis, inflammatory diseases, and metabolic diseases) (Figure 7). In that context, it has been found that SkM-released EVs have important paracrine actions that would participate in muscle physiology and may have an impact at the whole-body homeostasis.

As protein and miRNA compositions were different between ELV-MB and ELV-MT, it was postulated that they might be involved in the process of myogenesis. Indeed, it was shown that both ELV-MT protein and miRNA contents were transferred into proliferating myoblasts (Forterre et al., 2014a,b). Treatment of myoblasts with ELV-MT but not ELV-MB resulted in a decrease in myoblast proliferation and in the induction of early markers of differentiation (e.g., myogenin mRNA) (Forterre et al., 2014a). Bioinformatic analyses revealed that predicted target genes of differentially expressed miRNAs between ELV-MB and ELV-MT were mainly involved in the control of signaling pathways, and particularly the Wnt signaling pathway known to be regulated during myogenesis (Tanaka et al., 2011). Additionally, it was demonstrated that ELV-MT miRNAs were enriched in miRNA species targeting the 3’-UTR of *sirt* 1, a gene involved in muscle cell proliferation. Therefore, through the ELV route MT-ELV miRNAs could reduce the level of endogenous Sirt1 in myoblasts thus inducing cell differentiation (Forterre et al., 2014b). Furthermore, it was found that removing ELVs from bovine serum of cell culture, affected C2C12 proliferation (i.e., decrease of Cyclin D1 and Sirt1 mRNA levels) and committed cells to differentiate prematurely (induction of myogenin mRNA before myoblast fusion) (Aswad et al., 2016). In addition to observations that bovine ELVs can transfer specific signals to cells from unrelated species (mice C2C12), this result also suggests that ELVs in mammalian serum might have unsuspected functions during embryogenesis and in the regulation of cellular adaptations that lead to muscle hypertrophy, hyperplasia, and metaplasia. In line with this hypothesis, proliferation of C2C12 myoblasts is increased after 24 h of treatment with serum ELVs from mice suffering from Duchenne muscular dystrophy (*mdx* mice) compared with ELV-depleted serum (Matsuzaka et al., 2016), thus suggesting an important role of blood ELVs on the regulation of myogenesis and muscle mass.

In adult, myogenesis is stimulated in response to muscle damage and a role of SkM-ELVs was suggested in the control of muscle mass. In the context of hypertrophy, SkM satellite cells are activated to give rise to myogenic progenitor cells (MPCs) within the extracellular matrix (ECM) located around the fibers. It was found that MPCs secrete ELVs containing miR-206, which repressed the ribosomal binding protein 1, a master regulator of collagen biosynthesis, in fibrogenic recipient cells. This would prevent excessive ECM deposition for optimal muscle remodeling in response to hypertrophic stimuli (Fry et al., 2017). These data have provided insights into how skeletal stem and progenitor cells interact with other cell types to actively control their extracellular environments for tissue maintenance and adaptation. They have also provided a new mechanism to explain the fibrotic pathogenesis associated with other SkM alterations (i.e., muscular dystrophy, aging) in which satellite cell activity is perturbated and thus likely the release/composition of ELVs.

In the context of atrophy, treatment of C2C12 myotubes with dexamethasone for 48 h reduced the intracellular level of miR-23a but increased its ELV-MT abundance. As dexamethasone did not alter the number of ELVs released these data suggested that atrophy-inducing conditions lead to a selective packaging of miRNAs into ELV-MT (Hudson et al., 2014), thus changing the delivered message to recipient cells. In line with this hypothesis, it was demonstrated that after denervation, muscle fiber released a new population of ELVs enriched in miR-206 able to downregulate the expression of *Smarcd1* and *Runx2* proteins into NIH3T3 adipocytes (De Gasperi et al., 2017). It was also found that myoblasts incubated with ELVs released from myotubes pre-treated with H_2_O_2_ that mimics an oxidative stress, leads to a reduction in myotube diameter and to a stimulation of myoblast proliferation (Guescini et al., 2017). Similar results were obtained when myotubes were treated with a pro-inflammatory cytokine mixture of TNF-alpha and INF-gamma (Kim et al., 2018), suggesting that in addition to myogenesis, ELV-MT would also participate in the maintenance and regeneration of SkM mass following injuries.

In the context of metabolic diseases, *in vivo* data showed that palmitate-induced SkM insulin-resistance triggered the release of a new population of ELV-MT enriched in palmitate, in diet-induced obese mice (DIO) compared normal chow diet mice (NCD) (Aswad et al., 2014). It was demonstrated that palmitate contained in ELV-MT from DIO mice was transferred in recipient myotubes and resulted in the up-regulation of 240 genes involved in cell cycle and cellular adhesion. Among them, the pro-inflammatory cytokine IL-6 and the cell cycle regulator cyclin D1 were strongly upregulated, whereas Glut4 involved in glucose uptake was downregulated. In addition, muscle differentiation markers (myogenin, MyoD1, and CKMT2) were down-regulated showing that ELV-MT enriched in palmitate affected muscle phenotype. In addition to the release of a specific population of ELVs, mice fed with a palmitate-enriched diet released more ELV-MT (Aswad et al., 2014). It is likely that excessive concentration of circulating palmitate and saturated fatty acids associated with this specific high-fat diet had increased the concentration of intra-muscular ceramides that regulate ELV release from muscle (Matsuzaka et al., 2016). Interestingly, it has been found that intraperitoneal injections for 5–10 days of an inhibitor of ceramide synthesis improved muscle function and structure in *mdx* mice vs. untreated *mdx* animals (Matsuzaka et al., 2016). Although the composition and the biological action of SkM-ELVs from of these treated animals were not determined, it is a proof-of-concept that modulation of ELV release might be a therapeutic approach to regulate deleterious organ cross-talks associated with the transfer of deleterious EV cargos (e.g., the transfer of deleterious lipids during the development of metabolic diseases associated with high-fat diet).

Indeed, although communication between SkM and other tissues appears to be of importance in the development of various diseases, investigations to determine signals underlying these connections have been widely limited to the roles of cytokines. Recently, the possibility that SkM-ELVs could also act at systemic level has been studied in the context of type 2 diabetes associated with obesity (Jalabert et al., 2016). Intraperitoneal injections of fluorescent SkM-ELVs resulted in the fluorescent labeling of 9 organs in mice [i.e., brain, liver, heart, lungs, gastro-intestinal tract, spleen, pancreas, and kidney in addition to SkM (Aswad et al., 2014; Wiklander et al., 2015; Jalabert et al., 2016)]. ELVs collected from conditioned medium of SkM explants from DIO mice induced specific transcriptional signatures in pancreatic MIN6B1 recipient cells compared to SkM-ELVs from NCD mice. Microarray data analyses showed that the expressions of 460 mRNAs were significantly modulated including genes involved in immune responses known to be affected in pancreatic islets of diabetic patients (Esser et al., 2014; Jalabert et al., 2016). Additional bioinformatic analyses combined with *in vitro* experiments demonstrated that SkM-ELVs mediated the transfer of miRNAs from insulin-resistant muscles into MIN6B1 and affected the expression of proliferative genes such as *Ptch1* (Jalabert et al., 2016). These data suggested for the first time that SkM-released ELVs might transmit signals to the pancreas during the development of insulin-resistance. Because SkM-ELVs have no effect on insulin secretion, it would suggest that muscle-released ELVs would contribute to adaptations in beta cell mass occurring during insulin-resistance associated with obesity, at least in mice (Stamateris et al., 2013). As SkM is the unique organ for exercise, the variations of blood EV concentrations after physical activity have been studied (Frühbeis et al., 2015). The results indicate that a single bout of exhaustive exercise triggers the release of EVs with the size and marker profile of exosomes, which are cleared from the circulation during the early recovery period after cycling, but stay elevated after running exercise.

However, the clinical significance of these studies remains speculative until now and scientists have to be cautious not to over-interpret these data until the development of physiological models which would permit to follow EVs in blood vessels. Indeed, all results indicating a possible transfer of information between muscle cells and other tissues are based on *in vitro* data only. At current time, it not known whether SkM-ELVs could reach the blood circulation and have systemic actions. Recently, ELVs containing alpha-sarcoglycan (SGCA) a component of the dystrophin-glycoprotein complex involved in the stability of muscle fiber membranes, were found in human plasma (Guescini et al., 2015). These ELVs were also positive for CD81, TSG101 and miR-206, known to be highly expressed in SkM. Therefore, the authors of this study suggested that these SGCA positive ELVs might originate from SkM-ELVs and could be used as SkM liquid biopsies. Although we exclude this possibility, it has to be mentioned that SGCA and miR-206 are expressed in mainly other tissues (Mitchelson and Qin, 2015^2^) suggesting that the origin of the circulating pool of SGCA-positive ELVs is more complex and not only the result of SkM ELV secretion. In an attempt to follow muscle-released ELVs in blood, fluorescent ELVs were injected in the right *tibialis anterior* (TA) from mouse (Jalabert et al., 2016). Twenty-four hours after injection, fluorescence was detected in the left TA and in the right *quadriceps* too, suggesting again a paracrine-like action of muscle-released ELVs. Although these data strongly suggest that SkM can release ELV in blood, the generation of transgenic animals expressing GFP in SkM-released ELVs would help to quantify the impact of SkM-ELVs *in vivo* in the cross talks between SkM and key metabolic organs. Such model would also permit to identify all subtypes of EVs released from SkM cells in order to decipher their mode of biogenesis, and to understand how to modulate their release at the cellular level and to impact on their target tissues at the whole-body level.

## Challenges and Future Directions

The release of EVs with biological properties from muscle cells is an exciting new discovery given the importance of SkM in the regulation of glucose homeostasis and as organ of locomotion. Further studies are now required to determine the exact roles of these SkM-EVs at the whole-body level. In addition, it is important to identify the pathophysiologic conditions that influence their biogenesis, their compositions and fate. In the context of metabolic diseases or muscle-related pathologies, it is important to determine whether MVB biogenesis and EVs release might be new targets for therapy. Presently, this aspect is more or less considered by pharmaceutical companies to treat alterations of glucose/lipid metabolism, but the consequences on EV release/composition are not known, e.g., statins, used to reduce cholesterol level in patients and to prevent cardiovascular diseases target chemokines to MVBs in endothelial cells (Hol et al., 2012); metformin, the well-known antidiabetic drug affects the endosomal pathway by modulating pH levels in recycling endosomes and MVB (Kim et al., 2016; Kim and You, 2017). How these drugs affect EV release and compositions and participate in the side effects of the treatments is presently unknown, and has never been suggested. Beside to the use of drugs to modulate EV release, recent data suggest that EVs from healthy subjects might be used in the treatment of obesity, T2DM, and aging-associated metabolic disorders. Indeed, injections of adipose tissue macrophage-derived EVs isolated from lean mice improved glucose tolerance and insulin-sensitivity of diet-induced obese mice (Ying et al., 2017). Similarly, the injection of 3-month-old mice blood EVs to 18-month-old mice reversed the expression aging-associated biomarkers (Lee et al., 2018). The injection of MSC-derived EVs was found more efficient than the use of MSC in treating stroke (Chang et al., 2018). Based on these pre-clinical data, we can speculate that blood EVs isolated from athletes following exercise would transfer some of the beneficial systemic effects of exercise in patient suffering from metabolic disorders or SkM alterations (Frühbeis et al., 2015; Lovett et al., 2018). For example, it was demonstrated that endurance exercise induced the release of procoagulant MPs from blood cells in healthy subjects (Sossdorf et al., 2010). The authors of this study suggested that they would participate in the positive effect of exercise on vessel repair and wound healing, both affected in T2DM patients. Whether SkM-EVs from healthy subjects might be used to treat metabolic alterations or SkM dysregulation has never been envisaged.

## Author Contributions

SR drafted and wrote the manuscript. AF, MM, and KB critically advised and reviewed the manuscript.

### Conflict of Interest Statement

The authors declare that the research was conducted in the absence of any commercial or financial relationships that could be construed as a potential conflict of interest.

## Footnotes

^1^www.proteinatlas.org/ENSG00000110651-CD81/cell

^2^www.genecards.org
